# Evolution of the Global Burden of Viral Infections from Unsafe Medical Injections, 2000–2010

**DOI:** 10.1371/journal.pone.0099677

**Published:** 2014-06-09

**Authors:** Jacques Pépin, Claire Nour Abou Chakra, Eric Pépin, Vincent Nault, Louis Valiquette

**Affiliations:** Department of Microbiology and Infectious Diseases, Université de Sherbrooke, Sherbrooke, Québec, Canada; University of Athens, Medical School, Greece

## Abstract

**Background:**

In 2000, the World Health Organization estimated that, in developing and transitional countries, unsafe injections accounted for respectively 5%, 32% and 40% of new infections with HIV, hepatitis B virus (HBV) and hepatitis C virus (HCV). Safe injection campaigns were organized worldwide. The present study sought to measure the progress in reducing the transmission of these viruses through unsafe injections over the subsequent decade.

**Methods:**

A mass action model was updated, to recalculate the number of injection-related HIV, HCV and HBV infections acquired in 2000 and provide estimates for 2010. Data about the annual number of unsafe injections were updated. HIV prevalence in various regions in 2000 and 2010 were calculated from UNAIDS data. The ratio of HIV prevalence in healthcare settings compared to the general population was estimated from a literature review. Improved regional estimates of the prevalence of HCV seropositivity, HBsAg and HBeAg antigenemia were used for 2000 and 2010. For HIV and HCV, revised estimates of the probability of transmission per episode of unsafe injection were used, with low and high values allowing sensitivity analyses.

**Results:**

Despite a 13% population growth, there was a reduction of respectively 87% and 83% in the absolute numbers of HIV and HCV infections transmitted through injections. For HBV, the reduction was more marked (91%) due to the additional impact of vaccination. While injections-related cases had accounted for 4.6%–9.1% of newly acquired HIV infections in 2000, this proportion decreased to 0.7%–1.3% in 2010, when unsafe injections caused between 16,939 and 33,877 HIV infections, between 157,592 and 315,120 HCV infections, and 1,679,745 HBV infections.

**Conclusion:**

From 2000 to 2010, substantial progress was made in reducing the burden of HIV, HCV and HBV infections transmitted through injections. In some regions, their elimination might become a reasonable public health goal.

## Introduction

Injections made with a syringe and/or a needle previously used on another patient carry a risk of transmission of blood-borne viruses when equipment is re-used without adequate sterilization and correspond to an overwhelming majority of ‘unsafe injections', while use of multi-dose medication vials represents a smaller part of the problem. In 2000, the World Health Organization (WHO) estimated that, in developing and transitional countries, unsafe injections accounted for 5% of new HIV infections, 32% of new hepatitis B virus (HBV) infections and 40% of new hepatitis C virus (HCV) infections [1]–[3]. These estimates were based on a mass action model, in which the incidence of each blood-borne virus acquired from unsafe injections, ‘I_u_’, is a product of the size of the susceptible population, ‘p_s_’ (those not yet infected and, in the case of HBV, not yet vaccinated), the probability of transmission during an unsafe injection, ‘p_t_’, the probability that injection equipment is re-used, ‘p_r_’, the prevalence of the infection in the population, ‘p_v_’, and the number of injections performed per person-year, ‘n’, as follows: I_u_  =  p_s_ * [1-(1- p_t *_ p_r *_ p_v_)^n^].

Since then, the Safe Infection Global Network, ministries of health and other stakeholders have attempted to reduce the infectious risks associated with injections [4]. We reported elsewhere the changes from 2000 to 2010 in the number of unsafe injections per person-year, which decreased from 1.35 to 0.16 [5]. Here we attempted to quantify the evolution of the number of cases of injections-related HIV, HCV and HBV infections during that period. We first sought to recalculate the number of injection-related infections in 2000, using the same model but altering some parameters based on relevant information which has accrued since the previous work, and then we calculated the same outcomes for 2010, using updated epidemiological data. To allow comparisons, regions as defined in the 2000 Global Burden of Diseases (GBD) study were used (Table 1), excluding four high-income regions where unsafe injections are thought to be uncommon (North America/Cuba, Western Europe, Japan/Australia/New Zealand and other developed countries mostly in the Middle East) [1]–[3].

**Table 1:** Regions of the world (developing and transitional economies) as defined during the 2000 Global Burden of Diseases study.

**AFR D**	Algeria, Angola, Benin, Burkina Faso, Cameroon, Cape Verde, Chad, Comoros, Equatorial Guinea, Gabon, Gambia, Ghana, Guinea, Guinea-Bissau, Liberia, Madagascar, Mali, Mauritania, Mauritius, Niger, Nigeria, Sao Tome and Principe, Senegal, Seychelles, Sierra Leone, Togo
**AFR E**	Botswana, Burundi, Central African Republic, Congo, Côte d'Ivoire, Democratic Republic of the Congo, Eritrea, Ethiopia, Kenya, Lesotho, Malawi, Mozambique, Namibia, Rwanda, South Africa, Swaziland, Uganda, Tanzania, Zambia, Zimbabwe
**AMR B**	Antigua and Barbuda, Argentina, Bahamas, Barbados, Belize, Brazil, Chile, Colombia, Costa Rica, Dominica, Dominican Republic, El Salvador, Grenada, Guyana, Honduras, Jamaica, Mexico, Panama, Paraguay, Saint Kitts and Nevis, Saint Lucia, Saint Vincent and the Grenadines, Suriname, Trinidad and Tobago, Uruguay, Venezuela
**AMR D**	Bolivia, Ecuador, Guatemala, Haiti, Nicaragua, Peru
**EMR D**	Afghanistan, Djibouti, Egypt, Iraq, Morocco, Pakistan, Somalia, Sudan, Yemen
**EUR B**	Albania, Armenia, Azerbaijan, Bosnia and Herzegovina, Bulgaria, Georgia, Kyrgyzstan, Poland, Romania, Slovakia, Tajikistan, Macedonia, Turkey, Turkmenistan, Uzbekistan, Yugoslavia
**EUR C**	Belarus, Estonia, Hungary, Kazakhstan, Latvia, Lithuania, Republic of Moldova, Russian Federation, Ukraine
**SEAR B**	Indonesia, Sri Lanka, Thailand.
**SEAR D**	Bangladesh, Bhutan, Democratic People's Republic of Korea, India, Maldives, Myanmar, Nepal
**WPR B**	Cambodia, China, Cook Islands, Fiji, Kiribati, Lao, Malaysia, Marshall Islands, Micronesia, Mongolia, Nauru, Niue, Palau, Papua New Guinea, Philippines, Republic of Korea, Samoa, Solomon Islands, Tonga, Tuvalu, Vanuatu, Viet Nam

## Methods

### Number of unsafe injections per person per year

No change was made in the average number of unsafe injections per person per year in 2000 [1]–[3]. For 2010, in several regions there were reductions in unsafe injections, mostly through a lower proportion of re-use, and these figures were used for all three blood-borne viruses [5]. Given the lack of injections data for the three countries that constituted region SEAR B, extrapolations were made from India, Vietnam and Cambodia. Population figures were updated [6]. Changes in parameters specific for each virus are described below.

### Revised estimate of the probability of transmission per unsafe healthcare injection, p_t_

The probability of transmission of HIV, HCV and HBV per episode of unsafe healthcare injection cannot be measured directly, so that two proxies must be used: the risk of transmission during a needle stick injury in healthcare workers (HCW) and the risk of transmission per episode of needle/syringe sharing by injection drug users (IDU).

For HCV, the ‘p_t_’ for HCW injuries previously used (1.8%), generally accepted at the time, was overestimated because early studies had used unreliable diagnostic assays. A review published in 2002 estimated this ‘p_t_’ at 0.5% (59 infections after 11324 injuries) [7]. For HIV, the ‘p_t_’ for HCW injuries is generally estimated to be 0.32%, based on follow-up after 6202 exposures [8]–[12]. A 2006 meta-analysis estimated this risk at 0.24% [13]. The risk of transmission of HIV per episode of sharing of needles and/or syringes was estimated at between 0.63% and 1.57% [14]–[16].

When comparing unsafe injections to needle stick injuries, competing factors must be considered. Actions associated with an injection (inserting a needle deep into a muscle, and pushing its content with the plunger) may enhance the risk compared to HCW injuries, which are generally superficial. But on the other hand, one third of HCW injuries occur after a needle had been placed in the patient's vein (to draw blood, to insert an intravenous line, etc.) [17]–[18]. Most healthcare injections being made intramuscularly or subcutaneously, the amount of blood from the index patient that ends up in the needle/syringe is lower than when a HCW manipulated a needle deliberately inserted into a patient's vein. Furthermore, the ‘p_t_’ during unsafe injections must be lower than in IDU, among whom the potential transfer of viruses occurs from vein to vein.

Thus, the ‘p_t_’ of HIV (1.2%) and HCV (1.8%) per episode of contaminated healthcare injection used for 2000 were presumably overestimated [1], [3]. It is more prudent to use, for each virus, a low estimate, corresponding to the probability of transmission during a needle stick injury to a HCW, and a high estimate which probably should be not more than double the low one. For HIV, this corresponds to 0.32% and 0.64%, nearly identical to the 0.24%–0.65% proposed elsewhere in a meta-analysis [13], our high value for medical injections being close to the lower estimates (0.63%) of the transmission risk among IDU. For HCV, the same approach yields values of 0.5% and 1.0%.

The probability of HBV transmission during an unsafe injection had been assumed to be 6% for HBeAg-negative source patients and 30% for HBeAg-positive patients, in line with other estimates, and we used the same values given that this has not been studied further in recent years [1], [3], [19], [20]. However, it had been arbitrarily assumed that in most regions 20% of the HBsAg-positive individuals were HBeAg-positive, yielding an overall p_t_ = 10.8%, while elsewhere 50% were HBeAg-positive, for an overall p_t_ = 18% [1], [3]. We rather calculated region-specific values of ‘p_t_’, based on estimates of the proportion of HBeAg antigenemic individuals.

### Revised estimates of HIV prevalence in 2000 and estimates for 2010

UNAIDS revised retrospectively its measures of national HIV prevalence when Demographic and Health Surveys (DHS), with HIV testing on capillary blood, revealed that in several countries the prevalence (previously measured through surveys of pregnant women) had been overestimated because of an under-sampling of rural populations and an overestimate of the prevalence in men [21], [22]. The regional estimates of HIV prevalence for 2000 were recalculated, and those for 2010 were calculated the same way. From UNAIDS data for 2001 (revised figures) and 2009 [21], we extrapolated to 2000 and 2010, based on the mean annual changes in prevalence. Assuming that in most primary care settings children and adults are treated with the same pool of needles and syringes, the overall prevalence was calculated (and not merely among those aged 15–49 years).

### Estimation of HIV prevalence in healthcare settings

Previous calculations had used the HIV prevalence in the general population and assumed that prevalence among patients attending healthcare facilities was the same [1], [3]. However, HIV-infected patients develop symptoms for which they seek care and receive injections. Consequently, in certain healthcare settings, for instance patients hospitalized in a medical ward, the HIV prevalence is much higher than in the general population, as pointed out by Reid [23]. That effect, although less marked, is also present in primary care settings, even if a substantial fraction of their caseload corresponds to children, because HIV-infected children are also more likely to attend outpatient services than the seronegatives. Furthermore, in some primary care centers a substantial fraction of the caseload consists of patients with sexually transmitted infections, further enhancing HIV prevalence.

Furthermore, the propensity of HIV-infected patients to attend a health facility increases as the disease progresses and so does their viremia, hence their infectiousness. We assumed that this latter phenomenon was intrinsically tailored in within the estimates of the efficacy of transmission to HCW, and no further adjustments were made.

We reasoned that, worldwide, most injections (and most unsafe injections) are given to outpatients in primary care facilities: for-profit clinics (operated by physicians, nurses or unqualified personnel), governmental health centers, facilities run by non-profit organizations, outpatient departments of hospitals, etc. We assumed that, with regard to the syringes/needles used, patients treated in such facilities represent a single population (a mix of children and adults), rather than two distinct compartments each with their own pool of syringes/needles.

To identify relevant studies, Medline searches were performed (Appendix S1) and the US Census Bureau database was searched [24], seeking reports about patients in healthcare settings in developing/transitional countries published since 1995. The goal being to obtain measures of HIV prevalence among unselected, consecutive patients attending healthcare facilities, studies that represented obvious biases one way or the other were excluded, for instance measures among: i) inpatients, in which the HIV-infected would be much over-represented compared to outpatient settings; ii) patients presenting with conditions strongly associated with HIV infection (tuberculosis, pneumonia, etc.); iii) patients attending sexually transmitted diseases clinics or facilities for voluntary testing where the HIV-infected are over-represented; iv) antenatal clinic attendees and blood donors, since these visits are not prompted by ongoing symptoms. Furthermore, were excluded studies where the HIV status had been determined by a single test, studies with fewer than 200 participants, or with unavailable full text.

A total of 4052 titles and abstracts were scanned for full-text review and potential inclusion. Ultimately, 16 studies fulfilled all of the inclusion criteria and presented no exclusion criterion [25]–[41]. These measures of healthcare prevalence were compared with measures of HIV prevalence in the population of the same city or region. In some locations, this was possible through a DHS measure [22]. The prevalence in men and women combined was used, except for an all-women study in Uganda [34] for which the female prevalence was used as comparator. When the study population had been limited to some age groups the prevalence in age groups as close as possible was used as comparator. The comparator prevalence was adjusted for the interval that had elapsed between the study and the corresponding DHS, based on estimates for the variation in HIV prevalence between 2001 and 2009 [19]. For the paediatric studies, regional estimates made by the South African Department of Health for children aged 2–14 years were used as comparator [41]. For the other studies, our comparator prevalence was based on surveys of HIV among antenatal clinic attendees of the same location [24], generally available for the same year as the study itself. To translate this into a prevalence for the whole adult population, an adjustment took into consideration differences in prevalence between men and women, based on UNAIDS estimates in that particular country [21].

### Novel information on the prevalence of HCV and HBV in each region

Researchers recently estimated the prevalence of HCV seropositivity and HBsAg antigenemia in various regions in 1990 and 2005, for each sex and age stratum, based on a review of respectively 232 and 396 scientific papers and mathematical modelling [42], [43]. These estimates seemed more evidence-based than the empirical ones previously used [1]–[3]. We calculated the annual variation in the prevalence of HCV seropositivity and HBsAg antigenemia between 1990 and 2005, to extrapolate the prevalence in 2000 and 2010. To calculate the overall regional prevalence, the populations of each age stratum were used as weights [6]. As the data for 1990 and 2005 were presented along a revised classification of countries (GBD 2010), the latter was converted into prevalence for GBD 2000 regions, according to the proportions of each 2000 region that came from each 2010 region. No adjustment was made for a potentially higher prevalence in healthcare settings, which seems unlikely given that only a minority of HCV-infected and HBV-infected persons develop cirrhosis.

A similar exercise measured, in each region, the proportion of HBsAg-positive individuals who are HBeAg-positive, based on fewer publications [44]. Prevalence of HBeAg antigenemia among HBsAg-positive individuals was much higher in young children and decreased steadily with older age; geographic variations were modest.

In many low-income countries, HBV vaccine was introduced into the immunisation programme during the 2000–2010 decade, and the fraction of recipients of unsafe injections susceptible to iatrogenic HBV infection decreased progressively among children and adolescents. The proportions of the population of various age groups deemed non-susceptible through vaccination or natural infection were not altered for 2000, but had to be corrected for 2010. WHO collates data provided by member states concerning the proportion of infants who have received the third dose of HBV vaccine by the age of 12 months [45]. For each country, immunisation rates were calculated for two age strata, 0-4 years and 5–14 years, and translated into proportions of susceptibility (‘p_s_’) to HBV infection for each region, allowing for natural infections as well. For individuals older than 14 years, the same ‘p_s_’ as in the 2000 model [1], [3] were used.

## Results

### HIV prevalence

The revisions in HIV prevalence in the overall population had only a modest impact on the estimated regional prevalence for 2000 [1], [3]. For the prevalence in healthcare settings, thirteen studies contained data about adults, two presented paediatric data, and one had included both children and adults. Most studies had been performed in Africa, two in India and one in Haiti. HIV prevalence among study populations varied widely. The ratio between the prevalence among patients attending a healthcare facility and that in the comparator in each of 16 studies is shown in Supporting Information, Table S1. The means of these ratios was 2.48 for studies with adults, 2.69 for the paediatric studies, and 2.52 overall. This latter figure was multiplied by the prevalence in the overall population to derive the prevalence in healthcare settings, for each region, in 2000 and 2010 (Table 2). In 2010, this prevalence decreased in AFR E, and increased in EMR D and EUR C.

**Table 2:** Estimates of HIV prevalence (%) in the overall population and in healthcare settings, in 2000 and 2010.

	HIV prevalence in 2000		HIV prevalence in 2010	
Region	Overall	Healthcare settings	Overall	Healthcare settings
**AFR D**	1.46	3.68	1.36	3.42
**AFR E**	4.65	11.72	3.87	9.76
**AMR B**	0.31	0.79	0.29	0.73
**AMR D**	0.42	1.05	0.37	0.93
**EMR D**	0.05	0.12	0.14	0.34
**EUR B**	0.03	0.06	0.06	0.14
**EUR C**	0.28	0.71	0.63	1.58
**SEAR B**	0.23	0.57	0.26	0.65
**SEAR D**	0.23	0.58	0.18	0.45
**WPR B**	0.07	0.18	0.08	0.19

### Hepatitis C prevalence

The revised estimates of regional prevalence of HCV seropositivity for 2000 are displayed in Table 3 along with the data for 2010. Compared to the previously used data [1], [3], there was relatively little change in regional HCV prevalence for 2000. In 2010, prevalence increased in six regions.

**Table 3:** Estimates of HCV prevalence (%), in 2000 and 2010.

Region	Previous estimates for 2000^a^	Current estimates for 2000^b^	Estimates for 2010^b^
**AFR D**	2.63	3.30	2.58
**AFR E**	2.76	2.14	2.11
**AMR B**	1.51	1.44	1.60
**AMR D**	2.39	1.88	2.01
**EMR D**	5.53	3.21	3.44
**EUR B**	1.88	3.30	3.07
**EUR C**	2.45	2.62	3.20
**SEAR B**	2.89	2.23	1.91
**SEAR D**	1.84	3.08	3.90
**WPR B**	3.16	3.01	4.03

a Used in Hauri et al., and Hutin et al.^1,3^

b Derived from data available in Hanafiah et al.^42^

### Hepatitis B prevalence

The revised estimates of regional prevalence of HBsAg and HBeAg for 2000 are shown in Table 4. For the high-prevalence regions, the revised estimates of prevalence of HBsAg antigenemia were lower than before [1]. The revised proportions of the HBsAg-positives who were HBeAg antigenemic in 2000 were generally higher than in the original model, except for the two regions where this prevalence had been arbitrarily estimated at 50%. Since HBeAg antigenemia has a profound influence on the ‘p_t_’ for HBV, the regional ‘p_t_’ varied accordingly. Table 4 also displays the data for 2010. In all but one region, the prevalence of HBsAg antigenemia decreased. There was a modest reduction in the proportion of HBsAg-positive individuals who were HBeAg antigenemic.

**Table 4:** Prevalence (%) of HBsAg and HBeAg antigenemia, in 2000 and 2010.

	Previous estimates for 2000^a^		Current estimates for 2000^b^		Estimates for 2010^b^	
Region	Prevalence of HBsAg antigenemia	Proportion of HBsAg+ who are HBeAg+	Prevalence of HBsAg antigenemia	Proportion of HBsAg+ who are HBeAg+	Prevalence of HBsAg antigenemia	Proportion of HBsAg+ who are HBeAg+
**AFR D**	11.51	20	8.71	38.2	7.50	36.3
**AFR E**	11.84	20	6.93	39.9	6.46	34.7
**AMR B**	1.61	20	2.80	36.9	1.09	31.6
**AMR D**	2.01	20	4.48	37.8	3.71	32.6
**EMR D**	4.32	20	3.93	34.3	3.49	29.0
**EUR B**	5.51	20	4.06	33.1	3.17	28.6
**EUR C**	3.84	20	3.93	29.6	3.51	25.7
**SEAR B**	9.00	50	4.80	41.4	3.83	34.9
**SEAR D**	3.59	20	3.20	33.4	3.05	27.3
**WPR B**	11.83	50	7.01	38.3	7.28	32.1

a Used in Hauri et al., and Hutin et al.^1,3^

b Derived from data available in Ott et al.^43,44^

### Estimates of HIV infections transmitted through unsafe injections in 2000 and 2010

Table 5 shows the revised estimates of HIV infections transmitted through unsafe injections in 2000, based on the same model but with HIV prevalence in health care settings as ‘p_v_’ and with the two revised ‘p_t_’ values. Our higher estimate for 2000, based on p_t_ = 0.64%, yielded estimates similar to those presented initially [1], [3], with roughly a quarter of a million HIV infections acquired through unsafe injections. Naturally, the estimates with p_t_ = 0.32% yielded figures that were half the other measure. Based on previously mentioned assumptions, between 133,328 and 266,405 HIV infections were acquired worldwide through unsterile injections in 2000. Region SEAR D (mostly India) had represented more than half of the global number of injections-related cases of HIV, to a large extent because it was estimated that 75% of injections in SEAR D were made with re-used needles and syringes, based on a survey in India [1]–[3]. Despite a much higher ‘p_v_’, the contribution of sub-Saharan Africa was lower than SEAR D, because of fewer injections and less frequent re-use. Using UNAIDS revised data as denominators, in developing and transitional economies, between 4.6% and 9.1% of all new HIV infections in 2000 were caused by unsafe injections [21].

**Table 5:** HIV infections transmitted through unsafe injections, in 2000 and 2010.

	2000			2010	
Region	Previous estimates^a^	Revised estimates p_t_ = 0.32%	Revised estimates p_t_ = 0.64%	Estimates p_t_ = 0.32%	Estimates p_t_ = 0.64%
**AFR D**	18,317	13,641	27,274	1,734	3,468
**AFR E**	64,412	39,197	78,341	6,305	12,610
**AMR B**	305	214	429	622	1,243
**AMR D**	911	502	1,004	142	284
**EMR D**	2,210	2,340	4,678	1,704	3,407
**EUR B**	0	6	13	205	409
**EUR C**	1,526	1,734	3,467	1,284	2,568
**SEAR B**	6,260	3,382	6,762	574	1,148
**SEAR D**	156,663	68,005	135,821	3,020	6,039
**WPR B**	5,549	4,314	8,629	1,350	2,701
**World**	256,152	133,334	266,418	16,939	33,877

a Used in Hauri et al., and Hutin et al.^1,3^

For 2010, the main changes in parameters were in the number of unsafe injections per person per year [5] and the HIV prevalence in healthcare settings. The same two values of ‘p_t_’ were used. Between 16,939 and 33,877 HIV infections were acquired through unsafe injections worldwide (Table 5). Sub-Saharan Africa represented 48% of such cases, while the contribution of SEAR D decreased to 18%. Compared to 2000, the number of injections-related HIV infections acquired worldwide decreased by 87% in 2010, when between 0.7% and 1.3% of all new HIV infections were so acquired [21].

### Estimates of HCV infections transmitted through unsafe injections in 2000 and 2010

Table 6 shows the revised estimates of HCV infections transmitted through unsafe injections in 2000, based on the revised measures of prevalence and the two values of ‘p_t_’. We estimated that in 2000 between 952,111 and 1,867,904 HCV infections were injections-related. Again, the higher estimate for all ten regions was similar to the one generated previously [1], [3], even if the regional distribution varied. Table 6 also displays the results for 2010, using the same ‘p_t_’ values, the updated regional prevalence and the updated numbers of unsafe injections [5]. In 2010, between 157,592 and 315,120 HCV infections were acquired from unsafe injections, about one third of which occurred in EMR D and another third in WPR B.

**Table 6:** HCV infections transmitted through unsafe injections, in 2000 and 2010.

	2000			2010	
Region	Previous estimates^a^	Revised estimates p_t_ = 0.5%	Revised estimates p_t_ = 1.0%	Estimates p_t_ = 0.5%	Estimates p_t_ = 1.0%
**AFR D**	54,681	19,090	38,164	2,037	4,075
**AFR E**	54,131	11,642	23,281	2,218	4,437
**AMR B**	2,282	604	1,208	2,098	4,195
**AMR D**	6,304	1,374	2,748	472	944
**EMR D**	645,486	165,688	328,071	40,556	81,074
**EUR B**	2,110	1,047	2,094	8,258	16,512
**EUR C**	35,668	12,191	24,372	4,811	9,621
**SEAR B**	94,873	20,334	40,642	2,561	5,122
**SEAR D**	498,166	558,634	1,084,690	40,270	80,531
**WPR B**	608,200	161,508	322,633	54,311	108,609
**World**	2,001,901	952,111	1,867,904	157,592	315,120

a Used in Hauri et al., and Hutin et al.^1,3^

### Estimates of HBV infections transmitted through unsafe injections in 2000 and 2010

Table 7 displays the estimates of HBV infections transmitted through unsafe injections in 2000, using the same HBeAg-specific values of ‘p_t_’ applied on the revised estimates of the prevalence of HBsAg and HBeAg antigenemia. Although the regional figures varied along with modifications in the prevalence of antigenemia, the total for all ten regions was again similar to the one calculated previously for 2000, with 19,710,444 HBV infections acquired from injections. Table 7 also shows the results for 2010, based on the same values of ‘p_t_’ and the updated estimates of the number of unsafe injections and of the prevalence of HBsAg and HBeAg antigenemia in 2010 [5]. Compared to 2000, there was a 91% reduction in the number of injections-related HBV infections, to 1,679,745 new infections.

**Table 7:** HBV infections transmitted through unsafe injections, in 2000 and 2010.

Region	Previous estimates,^a^ 2000	Current estimates, 2000	Estimates for 2010
**AFR D**	639,498	675,362	52,282
**AFR E**	630,976	528,883	51,125
**AMR B**	14,118	33,743	28,969
**AMR D**	28,570	89,003	16,111
**EMR D**	2,533,443	3,684,450	500,198
**EUR B**	21,122	20,494	89,002
**EUR C**	193,636	251,548	52,124
**SEAR B**	942,038	448,601	22,508
**SEAR D**	8,019,210	10,188,564	400,985
**WPR B**	7,610,161	3,789,796	466,423
**World**	20,632,772	19,710,444	1,679,745

a Used in Hauri et al., and Hutin et al.^1,3^

## Discussion

The main finding of this study is that, between 2000 and 2010, there has been a reduction of respectively 87% and 83% in the estimated number of cases of HIV and HCV infections transmitted through unsafe injections. In the case of HBV, the reduction was more marked (91%) due to the additional impact of the rolling out of vaccination in most of the world.

We used the mathematical model developed previously [1], [3], because the main goal was to measure the relative reduction (2010 versus 2000) in injections-related HIV, HCV and HBV infections, but also because this model did not seem to be flawed, even if by definition all models are imperfect. A number of decisions about how to use it could be debated, however. First, random mixing between all age groups was assumed. That probably occurs in most private outpatient facilities, but less so in large hospitals. What proportion of injections worldwide is made through two distinct compartments, one for children and the other for adults remains unknown. Second, in our calculation of the relative prevalence of HIV in healthcare settings, inpatients data were excluded, lowering this estimate. What proportion of injections worldwide is given to outpatients versus inpatients remains unclear, and there might be a better compliance with single-use syringes and needles in hospital settings. Third, the values of ‘p_t_’ could be endlessly debated. Some authors argue that this probability is much higher than the values that we used [23], [47], but it does not seem plausible that transmission could be several fold more common during IM or SC unsafe medical injections than through IV injections of recreational drugs among addicts. Finally, potential biases in the measures of unsafe injections have been discussed elsewhere [5]. Apart from the latter, these sources of imprecision would be expected to have little impact on the measures of the relative reduction in the iatrogenic transmission of viruses over time.

Given that sampling variation and other imprecisions existed at various degrees for the five parameters used in the model, it was not possible to calculate confidence intervals around the absolute number of infections, and we elected to rather present sensitivity analyses for HIV and HCV based on two values of ‘p_t_’, the one parameter for which there was no direct measurement. HBV transmission during needle stick injuries has been little studied during the last 30 years using modern serological assays, precluding meaningful sensitivity analyses. In the future, model-based estimates could be complemented by the inclusion of children within the DHS of some countries, allowing a measurement of relatively recent non-sexual transmission of HIV and HCV, and of natural infections with HBV.

Of the three blood-borne viruses evaluated in the current study, HIV generally elicits most controversy [23], [46], [47]. There are reasons to believe that the revised measures for 2000 are improved compared to the prior version [1], [3]. The HIV prevalence in various regions of the world is better defined because it is now based, in many countries, on surveys of a representative sample of the nation's population. For the first time, an attempt was made to measure the relative prevalence of HIV in healthcare settings. And it seems likely that the two measures of ‘p_t_’, 0.32% and 0.64%, which now provide a sensitivity analysis, would be accepted by most experts. Ultimately, the number of injections-related HIV infections estimated previously for 2000 (256,152) [1], [3] was similar to our higher figure (266,405, if p_t_ = 0.64%), while our low estimate (133,328, if p_t_ = 0.32%) represented half of that measure. Despite the 13% population growth, the number of injections-related HIV infections decreased to only 16,939–33,877 in 2010, a remarkable public health achievement, and the fraction of new cases of HIV infection acquired through unsafe injections decreased to 0.7%–1.3% of the worldwide total of new infections in 2010, compared to 4.6%–9.1% ten years earlier. Most of this was driven by the reduction in the average number of unsafe injections, but decreasing HIV prevalence also impacted favourably in East and Southern Africa. We did not attempt to model the effect of the deployment of antiretrovirals on ‘p_t_’. This may need to be considered in the future, as the suppression of viremia lowers infectiousness but on the other hand prolongs survival, hence the duration of infectiousness.

The number of cases of HCV infections acquired from unsafe injections also declined substantially. Again our high estimate for 2000 was similar to the previous one [1], [3]. By 2010, the number of HCV infections from unsterile injections had dropped by 83%. The effect of the reduction in unsafe injections was attenuated by the population growth and the increasing prevalence in some densely populated regions [1]–[3]. The latter changes in HCV prevalence are likely multi-factorial: incomplete screening of blood donors, ongoing transmission among IDUs, and persistent transmission by parenteral modes other than injections. The long-term survival of most HCV-seropositive individuals also impacts on prevalence.

The progress with injections-related HBV infections was even more marked, at 91%. Several factors, attenuated only by the population growth, led to this reduction: fewer unsafe injections, lower prevalence of HBsAg and HBeAg antigenemia, and lower susceptibility to HBV through vaccination. Independently of any further progress in injection safety, this trend will continue as the immunised cohorts get older, producing direct and indirect effects. And as the HBsAg-positive subpopulation ages, it is also less prone to be HBeAg antigenemic, further reducing transmission.

Given this progress, the cost per additional case of injections-related HIV, HCV and HBV infections averted will increase, as is true for all disease control initiatives. We argue that these efforts should be maintained or expanded, even if more expensive, for two reasons. First, a moral imperative: iatrogenic infections with HIV, HCV and HBV are unacceptable, and go against a Hippocratic principle: ‘first, do no harm'. Second, as treatments against HIV and HCV are increasingly deployed in developing countries and transitional economies, incremental funding for the prevention of the remaining iatrogenic infections may generate savings. Elimination of these risks could become a reasonable goal in sub-Saharan Africa and Latin America. Such an achievement in Africa could remove half of the remaining burden of injections-related HIV infections worldwide.

However, other modes of iatrogenic transmission of blood-borne viruses, not covered by the current work, persist and will need to be addressed in the future. For instance, use of multi-dose medication vials, phlebotomies with re-used needles, dental care with improper sterilisation of instruments, unscreened transfusions, ritual scarifications and circumcisions performed by traditional practitioners all continue unabated, and should be included within ongoing efforts to reduce infectious risks for patients worldwide. Better measurement of such exposures and of their impact on viral dynamics is an essential first step, and the inclusion of children within demographic and health surveys could provide much needed data.

## Supporting Information

Table S1 **Estimation of the ratio of HIV prevalence in healthcare settings compared to the general population.**
(DOC)

Appendix S1 **Strategies to identify relevant publications for the comparison of HIV prevalence in healthcare setting with that of the general population.**
(DOC)
