# Supplementary material for: Evolution of the Global Burden of Viral Infections from Unsafe Medical Injections, 2000–2010
Source: PLoS One. 2014 Jun 9;9(6):e99677. doi: 10.1371/journal.pone.0099677 (PMC4049770; doi:10.1371/journal.pone.0099677)
Supplement: Table S1 — Estimation of the ratio of HIV prevalence in healthcare settings compared to the general population. (DOC) [file pone.0099677.s001.doc]

**Table S1.** Estimation of the ratio of HIV prevalence in healthcare settings compared to the general population.

| Healthcare population | | | | | | General population (comparator) | | | | | | | |
| --- | --- | --- | --- | --- | --- | --- | --- | --- | --- | --- | --- | --- | --- |
| Country | City/Region | Years | Age | Type of patients | HIV prevalence (%) | Source | City/Region | Years | HIV prevalence ANC (%) | ♀:♂ ratio, HIV in adults | % women in population | HIV prevalence comparator | Ratio prevalence healthcare vs comparator |
| Zambia | Lusaka | 1994-96 | Adults | Rheumatology outpatients | 71.6 | US CB | Lusaka | 1994-96 | 27.3 | 1.35 | 50.8 | 24.1 | 2.97 |
| Côte d’Ivoire | Abidjan | 1995-96 | Adults | Gynecology outpatients | 21.7 | US CB | Abdijan | 1995-96 | 14.0 | 1.28 | 49.1 | 12.2 | 1.78 |
| India | Vellore | 1999-2000 | Adults | Various outpatients | 0.7 | DHS | Tamil Nadu | 1998-99 | NR | NR | NR | 0.4 | 1.89 |
| Ghana | Agomanya | 1999 | All | Outpatients | 19.2 | US CB | Agomanya | 1999 | 6.4 | 1.27 | 50.0 | 5.7 | 3.36 |
| India | Andhra Pradesh | 2000 | Adults | Outpatients | 2.8 | DHS | Andhra Pradesh | 1998-99 | NR | NR | NR | 1.0 | 2.77 |
| Uganda | Gulu | 2000 | Adults | Acute fever | 21.6 | US CB | Gulu | 2000 | 15.3 | 1.44 | 51.3 | 13.0 | 1.66 |
| Malawi | Lilongwe | 2000-01 | Adults | Dermatology outpatients | 27.6 | US CB | Lilongwe | 2001 | 20.1 | 1.38 | 51.4 | 14.6 | 1.89 |
| Tanzania | Zanzibar | 2002 | Adults | Hospital outpatients | 2.7 | US CB | Zanzibar | 2002 | 1.1 | 1.50 | 51.0 | 0.9 | 2.89 |
| Uganda | 7 districts | 2002-04 | Adults | Malaria | 30.7 | US CB | Tororo district | 2003 | 6.9 | 1.44 | 51.3 | 6.0 | 5.12 |
| Uganda | Kampala | 2002-04 | 12-24 y | Female teenagers outpatients | 8.6 | DHS | Kampala | 2004-05 | NR | NR | NR | 7.2 | 1.20 |
| Haiti | Boucan Carré | 2003 | Adults | Primary care clinic | 3.1 | DHS | Région Centre | 2005-06 | NR | NR | NR | 1.8 | 1.75 |
| Zambia | Ndola | 2003-05 | 15-50 y | Malaria | 33.0 | DHS | Copperbelt | 2001-02, 2007 | NR | NR | NR | 18.3 | 1.80 |
| Uganda | Kampala | 2004-06 | Adults | Hospital OPD/casualty | 29.4 | DHS | Kampala | 2004-05 | NR | NR | NR | 8.2 | 3.61 |
| Uganda | 7 clinics | 2006-07 | ≥13 y | Suspected malaria | 11.2 | US CB | Arua, Jinja, Tororo | 2006-07 | 6.5 | 1.56 | 50.0 | 5.4 | 2.09 |
| Uganda | 7 districts | 2002-04 | 0-17 y | Malaria | 2.5 | UNAIDS | Uganda | 2003 | NR | NR | NR | 1.1 | 2.23 |
| South Africa | Free State | 2004 | 2-9 y | Primary health clinics | 13.9 | SA Dept Health | Free State | 2003, 2005 | NR | NR | NR | 4.1 | 3.42 |
| South Africa | Kwazulu-Natal, Limpopo | 2006-07 | 2-59 mo | Primary health clinics | 7.1 | SA Dept Health | Kwazulu-Natal, Limpopo | 2005, 2008 | NR | NR | NR | 2.9 | 2.41 |

Abbreviations: DHS, Demographic and Health Surveys; US CB, United States Census Bureau; OPD, Outpatients department; SA, South Africa; ANC, antenatal clinics; NR, Not relevant.
