# Supplementary material for: Evolution of the Global Burden of Viral Infections from Unsafe Medical Injections, 2000–2010
Source: PLoS One. 2014 Jun 9;9(6):e99677. doi: 10.1371/journal.pone.0099677 (PMC4049770; doi:10.1371/journal.pone.0099677)
Supplement: Appendix S1 — Strategies to identify relevant publications for the comparison of HIV prevalence in healthcare setting with that of the general population. (DOC) [file pone.0099677.s002.doc]

**Appendix S1.** Strategies to identify relevant publications for the comparison of HIV prevalence in healthcare setting with that of the general population.

**Strategy 1**

- EBM Reviews, Cochrane Database of Systematic Reviews 2005 to April 2012, Ovid MEDLINE(R) In-Process & Other Non-Indexed Citations and Ovid MEDLINE(R) 1946 to Present, and Pubmed:

Limits: human; date range: 01-01-1995 to 15-04-2012

Keywords: (HIV or HIV-1 or AIDS) and (health care or health setting or health center or health centre or hospital or primary or hospital) and (consecutive)

**Strategy 2**

Pubmed/ Limits: human; date range: 01-01-1995 to 15-04-2012

((HIV or HIV Seroprevalence or HIV Seropositivity or HIV-1) **and (outpatient))** not (prenatal or antenatal or pregnant or review or guideline or letter or editorial or trial or report or Australia or [OECD countries mentioned above])

Results were combined with those of:

((HIV or HIV Seroprevalence or HIV Seropositivity or HIV-1) AND **(outpatient or Primary Health Care or Health Services or Community Health Centers or Health Fairs or Health Facilities or Community Mental Health Services or Urban Health Services or Child Health Services or Adolescent Health Services or Mental Health Services or Rural Health Services or Suburban Health Services)** NOT (prenatal or antenatal or pregnant or review or guideline or letter or editorial or trial or report or Australia or [OECD countries mentioned above])

**Strategy 3**

Pubmed/ Limits: human; date range: 01-01-1995 to 15-04-2012

(HIV or HIV Seroprevalence or HIV Seropositivity or HIV-1) and (Outpatient) and: Africa/Asia/America NOT (United States OR Canada)/Europe NOT (Austria or [OECD European countries mentioned above])
